# Convection-Enhanced Delivery of Antiangiogenic Drugs and Liposomal Cytotoxic Drugs to Heterogeneous Brain Tumor for Combination Therapy

**DOI:** 10.3390/cancers14174177

**Published:** 2022-08-29

**Authors:** Ajay Bhandari, Kartikey Jaiswal, Anup Singh, Wenbo Zhan

**Affiliations:** 1 Department of Mechanical Engineering, Indian Institute of Technology (Indian School of Mines), Dhanbad 826004, India; 2 Centre for Biomedical Engineering, Indian Institute of Technology Delhi, New Delhi 110016, India; 3 Department of Biomedical Engineering, All India Institute of Medical Sciences, New Delhi 110029, India; 4 School of Engineering, King’s College, University of Aberdeen, Aberdeen AB24 3UE, UK

**Keywords:** antiangiogenesis, combination therapy, convection-enhanced delivery, DCE-MR imaging, heterogeneous tumor, mathematical model

## Abstract

**Simple Summary:**

Although developed anticancer drugs have shown desirable effects in preclinical trials, the clinical efficacy of chemotherapy against brain cancer remains disappointing. One of the important obstacles is the highly heterogeneous environment in tumors. This study aims to evaluate the performance of an emerging treatment using antiangiogenic and cytotoxic drugs. Our mathematical modelling confirms the advantage of this combination therapy in homogenizing the intratumoral environment for better drug delivery outcomes. In addition, the effects of local microvasculature and cell density on this therapy are also discussed. The results would contribute to the development of more effective treatments for brain cancer.

**Abstract:**

Although convection-enhanced delivery can successfully bypass the blood-brain barrier, its clinical performance remains disappointing. This is primarily attributed to the heterogeneous intratumoral environment, particularly the tumor microvasculature. This study investigates the combined convection-enhanced delivery of antiangiogenic drugs and liposomal cytotoxic drugs in a heterogeneous brain tumor environment using a transport-based mathematical model. The patient-specific 3D brain tumor geometry and the tumor’s heterogeneous tissue properties, including microvascular density, porosity and cell density, are extracted from dynamic contrast-enhanced magnetic resonance imaging data. Results show that antiangiogenic drugs can effectively reduce the tumor microvascular density. This change in tissue structure would inhibit the fluid loss from the blood to prevent drug concentration from dilution, and also reduce the drug loss by blood drainage. The comparisons between different dosing regimens demonstrate that the co-infusion of liposomal cytotoxic drugs and antiangiogenic drugs has the advantages of homogenizing drug distribution, increasing drug accumulation, and enlarging the volume where tumor cells can be effectively killed. The delivery outcomes are susceptible to the location of the infusion site. This combination treatment can be improved by infusing drugs at higher microvascular density sites. In contrast, infusion at a site with high cell density would lower the treatment effectiveness of the whole brain tumor. Results obtained from this study can deepen the understanding of this combination therapy and provide a reference for treatment design and optimization that can further improve survival and patient quality of life.

## 1. Introduction

Brain tumors have posed a unique health concern worldwide. Glioblastoma is the most aggressive primary brain cancer that remains incurable. The median survival is limited to 15 months even if the maximum treatment is applied [1]. Further, meningiomas are one of the most common intracranial tumors, with an annual incidence of about eight cases per 100,000 people [2,3]. The effectiveness of chemotherapy against brain cancers is disappointing. This can largely be attributed to the blood-brain barrier (BBB), which is impermeable to most anticancer drugs [4]. As an alternative to routine intravenous administration, convection-enhanced delivery (CED) has been developed to directly infuse anticancer drugs into tumor tissue [5]. Although the BBB can be bypassed mechanically upon CED, the delivery outcomes in clinical practice are still disappointing [6]. Inadequate drug accumulation and less-controllable drug spatial distribution are identified as the two significant limitations [7].

Drug delivery involves multiple physiological and physicochemical processes, determined by the transport properties of drugs, biological properties of tumors, and their interactions [8]. Solid tumors, particularly the microvasculature [9] and cell population [10] become highly heterogeneous as they grow. This heterogeneous nature can not only alter the intratumoral environment but also affect drug transport and accumulation, reducing the therapeutic efficacy [11]. In order to overcome this hindrance, antiangiogenic drugs are co-delivered with cytotoxic drugs to normalize the tumor microvasculature [12]. While feasibility has been demonstrated in animal experiments [13], drug delivery outcomes and efficacy in treating brain tumors in patients are unclear.

Mathematical modelling plays an increasingly important role in drug delivery research. Compared to in vivo experiments, this method is cost-effective for examining each drug delivery process individually, or in an integrated manner to explore the underlying drug delivery mechanisms and determine the impacts of influencing factors for optimization [14,15,16]. The modelling framework was first established in 1D in the pioneering studies [17,18,19], and further developed into 2D and 3D to predict the outcomes of various drug delivery methods and systems; these include intravenous administration [20,21], implantable wafer [22,23] and CED [24,25]. Moreover, great efforts [26] have been made to develop patient-specific modelling frameworks where the realistic intratumoral environment can be accommodated, particularly for CED [27,28,29]. The application of patient medical images can be the key to obtaining tissue properties. For instance, the nonuniform tumor microvasculature can be extracted from dynamic contrast-enhanced magnetic resonance (DCE-MR) images [30,31,32]. It is worth noting that previous modelling studies on CED mainly focus on the delivery of a single drug with the tumor’s heterogeneous nature ignored or simplified [33,34,35]. There is a dearth to simulate the combination therapy in the realistic tumor heterogeneous environment.

The current study applies an image-based transport model aimed to investigate the combined delivery of antiangiogenic and liposomal cytotoxic drugs to heterogeneous brain tumors upon CED. The 3D realistic geometry of a brain tumor and its heterogeneous characteristics are extracted from the patient’s DCE-MR images and imported into the mathematical model as input. The model covers the key processes in intracerebral drug delivery, including liposome release dynamics, transport in tissue interstitium by diffusion and convection, blood drainage, drug binding with proteins, cell uptake, and drug elimination due to physical degradation and bioreactions. The antiangiogenic effects on blood vessels and tissue interstitium are governed by a kinetic model. The treatment is evaluated in terms of the effective distribution volume, where the local drug concentration is sufficient to kill 90% of tumor cells.

## 2. Materials and Methods

### 2.1. Mathematical Model

Microvasculature networks can vary considerably in brain tumors depending on location and tumor growth stage. Given the dimensions of tumor and normal tissue (in cm) are usually about 3 orders higher than the distance between capillaries (in μm), the tumor and its holding tissue can therefore be treated as porous media in which the functions of capillaries are considered as distributed source terms [36]. Consequently, the incompressible, Newtonian interstitial fluid (ISF) flow is governed by the mass continuity equation and momentum conservation equation, which can be expressed as

(1) $$\nabla \cdot v=F_{\mathrm{BL}}$$

(2) $$\rho \frac{\partial v}{\partial t}+\rho \left(v\cdot \nabla v\right)=-\nabla p_{\mathrm{ISF}}+\mu \nabla^{2}v-\frac{\mu }{\kappa }v$$

where t is time. ρ is the interstitial fluid density, and μ is its dynamic viscosity. The interstitial fluid pressure (IFP) and velocity (IFV) are represented by $p_{\mathrm{ISF}}$ and v, respectively. κ is the tissue permeability that reflects the resistance of tissue to the interstitial fluid flow. $F_{\mathrm{BL}}$ is the flux of fluid gain from the blood (BL), driven by the effective transvascular pressure gradient. It can be calculated by Starling’s law, as

(3) $$F_{\mathrm{BL}}=L_{\mathrm{BL}}\frac{S_{\mathrm{BL}}}{V_{T}}\left[p_{\mathrm{BL}}-p_{\mathrm{ISF}}-\sigma_{\mathrm{BL}}\left(\pi_{\mathrm{BL}}-\pi_{\mathrm{ISF}}\right)\right]$$

where $L_{\mathrm{BL}}$ is the hydraulic conductivity of the capillary wall. $S_{\mathrm{BL}}/V_{T}=\varphi S_{\mathrm{BL},0}/V_{T}$ is the area of capillary surface per unit tissue volume, with the subscript 0 denoting the initial condition. φ is the scaling factor that reflects the antiangiogenic effect. $p_{\mathrm{BL}}$ is the blood pressure. $\sigma_{\mathrm{BL}}$ is the osmotic reflection coefficient for proteins in the blood. $\pi_{\mathrm{BL}}$ and $\pi_{\mathrm{ISF}}$ are the osmotic pressure of blood and interstitial fluid, respectively.

**Figure 1:**
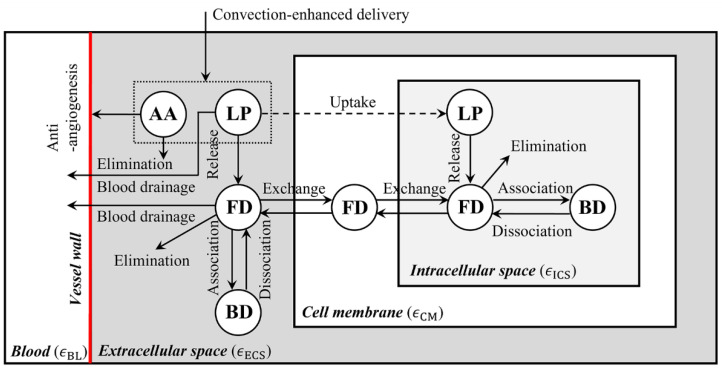
Drug transport in brain tumor after infusion. Liposomal cytotoxic drugs and antiangiogenic drugs are infused into the tumor ECS simultaneously. The released free cytotoxic drugs can cross the cell member to enter the cell interior; whereas liposome cell uptake strongly depends on the formulation, morphology and surrounding environment. As a result, some types of liposomes can enter cells efficiently, while others cannot cross the cell membrane. Therefore, this process is marked in a dashed line. Since there is a lack of a general model to govern this highly liposome-specific process due to its complexity, liposome cell uptake was ignored in several past studies [15,37]. AA—antiangiogenic drugs, LP—liposome-encapsulated cytotoxic drugs, FD—free cytotoxic drugs, and BD—cytotoxic drugs that bind with proteins.

A brain, including the embedded tumor, consists of the microvasculature network and tissue; the latter can be further divided into the extracellular space (ECS), cell membrane (CM) and intracellular space (ICS). The transport of infused drugs between these compartments is schematically shown in Figure 1, where the letters LP, FD and BD stand for the liposome-encapsulated drugs, released free drugs and the drugs that bind with proteins, respectively. By assuming the liposomes are stable before entering a tumor, the concentration of liposome-encapsulated drugs ($C_{\mathrm{LP},\mathrm{ECS}}$) can be described by

(4) $$\frac{\partial \epsilon_{\mathrm{ECS}}C_{\mathrm{LP},\mathrm{ECS}}}{\partial t}=D_{\mathrm{LP},\mathrm{ECS}}\nabla^{2}\left(\epsilon_{\mathrm{ECS}}C_{\mathrm{LP},\mathrm{ECS}}\right)-v\cdot \nabla \left(\epsilon_{\mathrm{ECS}}C_{\mathrm{LP},\mathrm{ECS}}\right)-k_{\mathrm{rel},\mathrm{ECS}}\epsilon_{\mathrm{ECS}}C_{\mathrm{LP},\mathrm{ECS}}\;-k_{\mathrm{LP},b}\epsilon_{\mathrm{ECS}}C_{\mathrm{LP},\mathrm{ECS}}-F_{\mathrm{BL}}\epsilon_{\mathrm{ECS}}C_{\mathrm{LP},\mathrm{ECS}}$$

where $\epsilon_{\mathrm{ECS}}$ is the volume fraction of tissue extracellular space. $D_{\mathrm{LP},\mathrm{ECS}}$ is the diffusivity of liposomes in tissue ECS. $k_{\mathrm{rel}}$ is the drug release rate from liposomes. $k_{\mathrm{LP},b}=P_{\mathrm{TV},\mathrm{LP}}\frac{S_{\mathrm{BL}}}{V_{T}}$ is the loss rate of liposomes to the blood, where $P_{\mathrm{TV},\mathrm{LP}}$ is the liposome transvascular permeability.

The concentration of free drugs ($C_{\mathrm{FD},T}$) and bound drugs ($C_{\mathrm{BD},T}$) in the tissues can be accounted by

(5) $$\begin{array}{c} \left(1-\epsilon_{\mathrm{BL}}\right)C_{\mathrm{FD},T}=\epsilon_{\mathrm{ECS}}C_{\mathrm{FD},\mathrm{ECS}}+\epsilon_{\mathrm{CM}}C_{\mathrm{FD},\mathrm{CM}}+\epsilon_{\mathrm{ICS}}C_{\mathrm{FD},\mathrm{ICS}}\\ \left(1-\epsilon_{\mathrm{BL}}\right)C_{\mathrm{BD},T}=\epsilon_{\mathrm{ECS}}C_{\mathrm{BD},\mathrm{ECS}}+\epsilon_{\mathrm{CM}}C_{\mathrm{BD},\mathrm{CM}}+\epsilon_{\mathrm{ICS}}C_{\mathrm{BD},\mathrm{ICS}} \end{array}$$

where $\epsilon_{\mathrm{BL}}$, $\epsilon_{\mathrm{CM}}$ and $\epsilon_{\mathrm{ICS}}$ are the volume fractions of plasma, cell membrane and intracellular space, respectively. $C_{\mathrm{BD},\mathrm{CM}}$ is zero as no drugs would be eliminated or bound on the cell membrane [22].

The accumulation of free drugs in tissue is determined by multiple factors, including the dynamic release from liposomes, transport by convection and diffusion, blood drainage, drug elimination due to physical degradation and bioreaction, binding with proteins and cell uptake. Based on the law of conservation of mass, the availability of free drugs in tissue is governed by

(6) $$\begin{array}{ll} \frac{\partial \left(1-\epsilon_{\mathrm{BL}}\right)C_{\mathrm{FD},T}}{\partial t} & =D_{\mathrm{FD},\mathrm{ECS}}\nabla^{2}\left(\epsilon_{\mathrm{ECS}}C_{\mathrm{FD},\mathrm{ECS}}\right)-\nabla \cdot \left(\epsilon_{\mathrm{ECS}}C_{\mathrm{FD},\mathrm{ECS}}v\right)\\ & -\epsilon_{\mathrm{ECS}}\left(k_{\mathrm{FD},b}+k_{\mathrm{FD},e}\right)C_{\mathrm{FD},\mathrm{ECS}}-k_{\mathrm{FD},e}\epsilon_{\mathrm{ICS}}C_{\mathrm{FD},\mathrm{ICS}}\\ & +k_{\mathrm{rel},\mathrm{ECS}}\epsilon_{\mathrm{ECS}}C_{\mathrm{LP},\mathrm{ECS}}-\frac{\partial \left(1-\epsilon_{\mathrm{BL}}\right)C_{\mathrm{BD},T}}{\partial t} \end{array}$$

where $D_{\mathrm{FD},\mathrm{ECS}}$ is the diffusivity of free drugs in tissue ECS. $k_{\mathrm{FD},b}$ is the loss rate of free drugs to the blood, calculated by $k_{\mathrm{FD},b}=P_{\mathrm{TV},\mathrm{FD}}\frac{S_{\mathrm{BL}}}{V_{T}}$. $P_{\mathrm{TV},\mathrm{FD}}$ is transvascular permeability of free drugs. $k_{\mathrm{FD},e}$ is the elimination rate of free drugs, integrating the contributions of physical degradation and bioreaction. Two assumptions are further introduced: (a) The concentration of free drugs and bound drugs are linearly correlated in the tissue ECS and ICS ($C_{\mathrm{BD},\mathrm{ECS}}=K_{\mathrm{BC}}C_{\mathrm{FD},\mathrm{ECS}}$; $C_{\mathrm{BD},\mathrm{ICS}}=K_{\mathrm{BC}}C_{\mathrm{FD},\mathrm{ICS}}$) [23,38]; (b) Dynamic equilibrium is reached for the concentrations of free drugs between the tissue compartments ($C_{\mathrm{FD},\mathrm{ICS}}=H_{\mathrm{IE}}C_{\mathrm{FD},\mathrm{ECS}}$; $C_{\mathrm{FD},\mathrm{CM}}=H_{\mathrm{CE}}C_{\mathrm{FD},\mathrm{ECS}}$) [39,40]. Therefore, Equation (6) can be rewritten as

(7) $$\frac{\partial \omega C_{\mathrm{FD},\mathrm{ECS}}}{\partial t}=D_{\mathrm{FD},\mathrm{ECS}}\nabla^{2}\left(\epsilon_{\mathrm{ECS}}C_{\mathrm{FD},\mathrm{ECS}}\right)-v\cdot \nabla \left(\epsilon_{\mathrm{ECS}}C_{\mathrm{FD},\mathrm{ECS}}\right)+k_{\mathrm{rel},\mathrm{ECS}}\epsilon_{\mathrm{ECS}}C_{\mathrm{LP},\mathrm{ECS}}\;-\left[\epsilon_{\mathrm{ECS}}\;k_{\mathrm{FD},b}+\left(\epsilon_{\mathrm{ECS}}+\epsilon_{\mathrm{ICS}}\right)k_{\mathrm{FD},e}+\epsilon_{\mathrm{ECS}}F_{\mathrm{BL}}\right]C_{\mathrm{FD},\mathrm{ECS}}$$

where $\omega =\epsilon_{\mathrm{ECS}}\left(1+K_{\mathrm{BC}}\right)+\epsilon_{\mathrm{ICS}}H_{\mathrm{IE}}\left(1+K_{\mathrm{BC}}\right)+\epsilon_{\mathrm{CM}}H_{\mathrm{CE}}$ is a parameter determined by the properties of the drug and tissues.

The concentration of antiangiogenic drugs in the tissue ECS depends on the convective and diffusive transport, and elimination, as

(8) $$\frac{\partial \epsilon_{\mathrm{ECS}}C_{\mathrm{AA},\mathrm{ECS}}}{\partial t}=D_{\mathrm{AA},\mathrm{ECS}}\nabla^{2}\left(\epsilon_{\mathrm{ECS}}C_{\mathrm{AA},\mathrm{ECS}}\right)-v\cdot \nabla \left(\epsilon_{\mathrm{ECS}}C_{\mathrm{AA},\mathrm{ECS}}\right)-k_{\mathrm{AA},e}\epsilon_{\mathrm{ECS}}C_{\mathrm{AA},\mathrm{ECS}}\;-F_{\mathrm{BL}}\epsilon_{\mathrm{ECS}}C_{\mathrm{AA},\mathrm{ECS}}$$

where $D_{\mathrm{AA},\mathrm{ECS}}$ is the diffusivity of antiangiogenic drugs in the tissue ECS. $k_{\mathrm{AA},e}$ is its elimination rate.

The antiangiogenic effect can be described by

(9) $$\frac{d\varphi }{dt}=\varphi \left(\alpha +\beta \varphi +\gamma \varphi^{2}\right)-k_{a}\varphi C_{\mathrm{AA},\mathrm{ECS}}$$

in which φ is the scaling factor for microvasculature density. $k_{a}$ is the antiangiogenic rate. α, β and γ are applied to consider the natural angiogenesis of the tissue.

### 2.2. MR Imaging and Processing Protocol

MR scan of two brain tumor patients was performed on a 3.0-Tesla Ingenia MRI scanner (Philips Healthcare, Amsterdam, The Netherlands) at Fortis Memorial Research Institute, Gurugram, India. MR imaging protocol included a standard protocol for brain tumor patients along with DCE MRI or T1-Perfusion MRI. DCE MR images of the brain were acquired using the 3D fast field echo (T1-FFE) sequence before, during and after contrast injection. In the current study, a systemic infusion of the contrast agent Dotarem (Gadoterate meglumine, Guerbet, France) with a dose of 0.1 mmol/kg body weight at an infusion rate of 3.0 mL/s was used. The key imaging parameters are summarised in Table 1.

All procedures performed in this study involving human participants were in accordance with the ethical standards of the Institutional Review Board (IRB No. 2020-001-19-28) and with the 1964 Helsinki declaration and its later amendments or comparable ethical standards. The demographic characteristics for the two exemplary patients are given in Table 2, where a pre-2016 classification is applied.

#### 2.2.1. 3D Reconstruction of Brain Tumor

**Figure 2:**
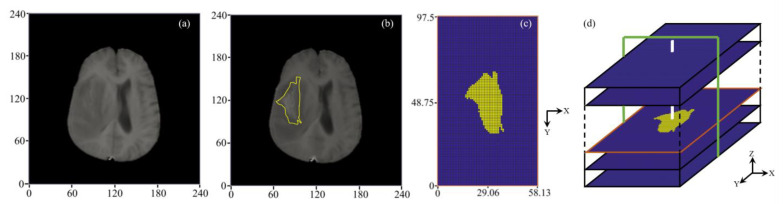
Model geometry. (**a**) Pre-contrast, and (**b**) post-contrast MR image of a representative slice with a yellow outline showing tumor boundary. (**c**) The segmentation result, along with CFD mesh where the tumor and its holding tissue are marked in yellow and blue, respectively. (**d**) Schematical diagram showing the 3D geometry reconstruction. Drugs are infused through the catheter marked in white color. Modelling results will be presented in the X-Z plane along the catheter track in the following sections, marked in green color.

A representative DCE-MR image slice and reconstructed 3D configuration are represented in Figure 2. The brain tumor is segmented from its holding tissue based on the local signal intensity on each image slice. These segmentation results are then stacked along the MR scan direction to reconstruct the brain tumor in 3D. The tumor equivalent radius and volume are measured as 16 mm and 5461 ${\mathrm{mm}}^{3}$, respectively. Given this study is focused on the drug transport in the brain tumor rather than the whole brain, a rectangular volume [41,42] of interest with the dimension of $58\times 97\times 72\;{\mathrm{mm}}^{3}$ is generated to fully enclose the brain tumor.

The final computational mesh consists of 82,944 uniform structured elements, obtained after the mesh-independence test [42]. The dimension of each element is 0.9×0.9×6.0 mm. Drugs are infused into the brain tumor through the catheter with a dimension of 0.9 mm, as shown in Figure 2c.

#### 2.2.2. Extraction of Tumor Heterogenous Properties

The quantitative analysis of DCE-MRI data is performed by computing the concentration of contrast agent from the signal intensity of the DCE-MR images on a voxel-by-voxel basis. This is based on the spoiled gradient recalled echo (SPGR)/FFE equation that is represented as [43]

(10) $$\frac{I\left(t\right)}{I\left(0\right)}=k_{0}\exp\left[-TER_{2}C_{\mathrm{Gd},\mathrm{ECS}}\left(t\right)\right]\frac{1-\exp\left\{-TR\left[T_{10}^{-1}+R_{1}C_{\mathrm{Gd},\mathrm{ECS}}\left(t\right)\right]\right\}}{1-\cos\left(\theta \right)\exp\left\{-TR\left[T_{10}^{-1}+R_{1}C_{\mathrm{Gd},\mathrm{ECS}}\left(t\right)\right]\right\}}$$

(11) $$k_{0}=\frac{1-\cos\left(\theta \right)\exp\left(-TRT_{10}^{-1}\right)}{1-\exp\left(-TRT_{10}^{-1}\right)}$$

where I(0) is the signal intensity when no contrast agent was given. I(t) is the signal intensity of the contrast agent at a particular time point. $C_{\mathrm{Gd},\mathrm{ECS}}\left(t\right)$ is the contrast agent concentration in the extracellular space ($\mathrm{mmol}\cdot L^{-1}$). The longitudinal relaxivity ($R_{1}$) and transverse relaxivity ($R_{2}$) of Dotarem contrast agent in the plasma are $3.5\;{\mathrm{mmol}}^{-1}s^{-1}L$ and $4.9\;{\mathrm{mmol}}^{-1}s^{-1}L$, respectively. The procedure developed in Ref [43] is adopted to calculate the pre-contrast $T_{1}\left(T_{10}\right)$ using the three fast spin-echo (FSE) images, including T1-weighted images (TR/TE=360/10 ms), T2-weighted images (TR/TE=3500/90 ms) and proton density (PD)-weighted images (TR/TE=3500/7.2 ms). The rest imaging parameters for acquisition of T1-weighted, T2-weighted and PD-weighted images were the same as for T1-perfusion MR images, as summarised in Table 1.

The Leaky Tracer Kinetic Model (LTKM) [44] is applied to calculate the tissue perfusion parameters at each image voxel by fitting to the local contrast agent concentrations. Compared to the General Tracer Kinetic Model (GTKM), it is favored to analyze DCE-MRI data obtained in a short acquisition time [45,46], as the data used in this study (2 min). This is because GTKM requires relatively longer DCE-MRI data to stabilize the contrast agent’s concentration and accurately determine the porosity. This is not always possible in the case of humans because of many reasons, such as prolonged scan time and distortion of the image caused by the patient’s movement during scan time. The LTKM is expressed as

(12) $$C_{\mathrm{Gd}.\mathrm{ECS}}\left(t\right)=\epsilon_{\mathrm{BL}}C_{\mathrm{Gd},\mathrm{BL}}\left(t\right)+K^{\mathrm{trans}}\int_{0}^{t}C_{\mathrm{Gd},\mathrm{BL}}\left(\tau \right)e^{\frac{K^{\mathrm{trans}}}{\epsilon_{\mathrm{ECS}}}\left(\tau -t\right)}d\tau \;+\lambda^{\mathrm{tr}}\int_{0}^{t}C_{\mathrm{Gd},\mathrm{BL}}\left(\tau \right)d\tau$$

where $K^{\mathrm{trans}}$ is transvascular transport rate between plasma and extracellular space. $\lambda^{\mathrm{tr}}$ is the volume transfer constant between plasma and leakage space. $C_{\mathrm{Gd},\mathrm{BL}}$ as the arterial input function stands for the concentration of contrast agent in the blood, which can be obtained from the images following the procedure in Ref [47]. Readers are referred to the identified references for details of the image processing protocols.

### 2.3. Model Parameters

Given that the treatment period is much shorter than the tumor growth rate, the drug transport properties and tissue biological properties are treated as constants. Bevacizumab (BEV) and temozolomide (TMZ), as the typical antiangiogenic drug and cytotoxic drug, respectively, are selected in this study. The baseline values of model parameters are summarized in Table 3 and Table 4 for tissues and drugs, respectively. Model parameters stranding for the heterogeneous tissue properties are extracted from DCE-MRI and mapped to each voxel of the 3D model geometry based on the coordinates. The MR images also consist of voxels present outside the brain, which belong neither to the tumor nor its surrounding tissue, i.e., exterior voxels corresponding to air. The model parameters are set as zero for these voxels [42,48].

#### 2.3.1. Volume Fraction (ϵ)

**Figure 3:**
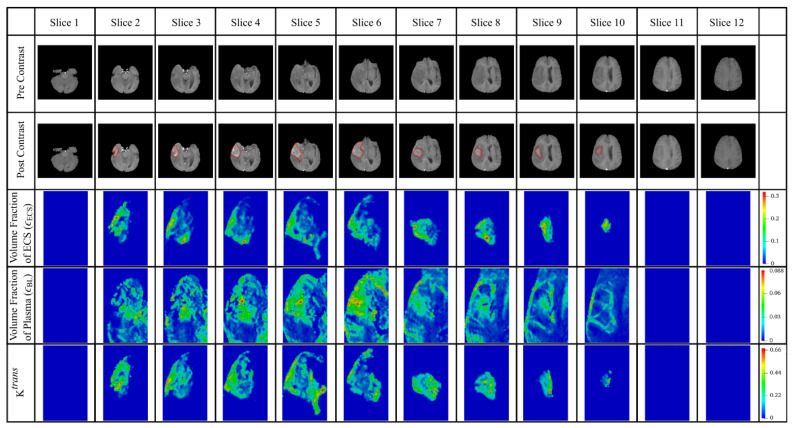
Tissue heterogeneous properties extracted from patient DCE-MR images. The interface between the brain tumor and its holding tissue is marked in red in the post-contrast images.

The initial volume fraction of plasma ($\epsilon_{\mathrm{BL},0}$) and extracellular space ($\epsilon_{\mathrm{ECS},0}$) can be directly obtained from DCE-MR data as described in Section 2.2.2. Results of each image slice are given in Figure 3, where the tumor region presents a higher volume fraction of ECS than normal tissue. Since the ratio of $\epsilon_{\mathrm{CM}}/\epsilon_{\mathrm{ICS}}$ is around 0.154 and 0.188 [22,65] for tumor cells and normal cells, respectively, the initial volume fraction of cell membrane ($\epsilon_{\mathrm{CM},0}$) and intracellular space ($\epsilon_{\mathrm{ICS},0}$) at each image voxel can be calculated by using

(13) $$\epsilon_{\mathrm{BL}}+\epsilon_{\mathrm{ECS}}+\epsilon_{\mathrm{CM}}+\epsilon_{\mathrm{ICS}}=1$$

The antiangiogenic drugs, which are continuously infused into the brain tumor, can reduce the surface area of microvasculature over time by the scaling factor φ, as $S_{\mathrm{BL}}/V_{T}=\varphi S_{\mathrm{BL},0}/V_{T}$. $S_{\mathrm{BL}}$ is the surface area of the microvasculature wall, defined as $S_{\mathrm{BL}}=2\pi rl$, where r and l are the mean radius and mean length of the blood vessels, respectively. Studies using microscopy imaging demonstrated that antiangiogenic drugs can not only successfully lead to the shrinkage of microvasculature, but also reduce the blood vessel length [66,67]. However, given there is no kinetic model to describe the relationship between the concentration of antiangiogenic drugs and the radius and length of blood vessels, it is further assumed that only the vessel radius reduces in response to the antiangiogenic drugs. Given the tissue volume remains constant, $r=\varphi r_{0}.$ Moreover, since the volume fraction of plasma is defined as $\epsilon_{\mathrm{BL}}=V_{\mathrm{BL}}/V_{T}=\pi r^{2}l/V_{T}$, the antiangiogenic effect on the volume fraction of plasma ($\epsilon_{\mathrm{BL}}$) can be expressed as

(14) $$\epsilon_{\mathrm{BL}}=\varphi^{2}\epsilon_{\mathrm{BL},0}$$

The parameter, volume fraction of plasma ($\epsilon_{\mathrm{BL}}$) is applied as the measure of microvascular density in this study.

The value of $\epsilon_{\mathrm{ECS}}$ can then be updated using Equation (13) by treating the volume fractions of the cell membrane and intracellular space as constants.

#### 2.3.2. Microvasculature Surface Area per Unit Tissue Volume ($S_{\mathrm{BL}}/V_{T}$)

Microvasculature surface becomes highly nonuniform as tumors grow. The ratio of $K^{\mathrm{trans}}$ (shown in Figure 3 for each slice) at each image voxel to the average value for the entire tumor region is then used to calculate the initial values for microvasculature density ($S_{\mathrm{BL},0}/V_{T}$) by scaling with the standard value [68], ${\left(S_{\mathrm{BL}}/V_{T}\right)}_{b}$ from the literature, as

(15) $$\frac{S_{\mathrm{BL},0}}{V_{T}}=\frac{K^{\mathrm{trans}}}{K_{\mathrm{avg}}^{\mathrm{trans}}}{\left(\frac{S_{\mathrm{BL}}}{V_{T}}\right)}_{b}$$

#### 2.3.3. Infusion Settings

Infusion settings including the infusion rate ($R_{\mathrm{in}}$), infusion duration ($T_{\mathrm{in}}$) and infusion concentration ($C_{\mathrm{in}})$ are the factors that can be precisely controlled in clinical practice, directly determining the drug dose for administration. The infusion rate is usually kept in the range of 0.5~10.0 μL/min [69,70] to provide effective drug administration, while avoiding potential tissue damage. Moreover, it is found that the CED infusion catheter can be left indwelling for several days when the infusion rate is kept below 5.0 μL/min [7]. Since drugs were infused at the rate of 5.0 μL/min for 2~5 days in the clinical trials [69], the CED infusion is conducted at the rate of 3.0 μL/min in this study, lasting for 3 days. Given BEV and TMZ were infused at the concentrations of $7.26\times 10^{-5}\;M$ [71] and $5.15\times 10^{-3}\;M$ [72], respectively, in the preclinical experiments, the same settings are applied in the following simulations.

### 2.4. Numerical Methods

The governing equations are implemented into a finite volume method-based Computational Fluid Dynamics open-source code package, OpenFOAM, to generate numerical solutions. The transient term is discretized with the first-order Euler scheme. The Gauss linear, linear interpolation and Gauss linear upwind schemes are employed to discretize the gradient, Laplacian and divergence terms in the governing equations, respectively. Fluid pressure and velocity correction are linked by the pressure implicit splitting of operators (PISO) algorithm. The residual tolerances are set as $1.0\times 10^{-6}$ for the interstitial fluid transport model and $1.0\times 10^{-8}$ for the drug transport model, respectively, to control the modelling solution convergence. A fixed time step of $1.0\times 10^{-3}\;s$ is selected after the time step-independence test. The governing equations of interstitial fluid flow are solved under the condition of no infusion in the first place to generate a steady-state solution, which represents the hydraulic environment in the tissues before the CED infusion takes place. This solution is then applied at time zero for simulating the interstitial fluid flow and drug transport upon CED infusion in a transient manner. The initial concentrations of BEV, liposomal TMZ and free TMZ are set as zero in the entire domain.

The numerical simulations are performed using a 64-bit Intel(R) Core i7-10700 processor (Clock speed: 2.90 GHz), eight cores with 32 GB RAM. The total computational time involved in solving the fluid flow and drug transport equations is approximately 1.5 h.

### 2.5. Boundary Conditions

The external surface of normal tissue is assumed to be fixed with zero gauge pressure and drug flux, as it is the source-sink-driven flow in tissue [41,42]. The continuous condition is employed at the tumor-normal tissue interface. The catheter wall is assumed to be rigid with zero slip or flux. The fixed infusion rate is specified at the infusion site, where the flux of free drugs is zero. The concentration of liposomes at the infusion site remains constant over the entire infusion duration for drug administration.

### 2.6. Qualification of Delivery Outcomes

The delivery outcomes of this combination therapy under different regimens are evaluated by the following qualification indexes from the perspectives of drug accumulation, drug distribution and treatment effectiveness.

#### 2.6.1. Spatial-Averaged Concentration

The drug concentration directly reflects the amount of drugs accumulating in the tissue. It is determined by the aforementioned drug transport processes and varies throughout the brain tumor and its holding tissue. Spatial-averaged concentration ($C_{\mathrm{FD},\mathrm{ECS},\mathrm{avg}}$) is applied to examine the drug accumulation in the entire tissue, which is expressed as

(16) $$C_{\mathrm{FD},\mathrm{ECS},\mathrm{avg}}=\frac{\sum^{\;}C_{\mathrm{FD},\mathrm{ECS},i}V_{\mathrm{ECS},i}}{\sum^{\;}V_{\mathrm{ECS},i}}=\frac{\sum^{\;}C_{\mathrm{FD},\mathrm{ECS},i}\epsilon_{\mathrm{ECS},i}V_{T,i}}{V_{\mathrm{ECS},T}}$$

where $C_{\mathrm{FD}\;\mathrm{ECS},i}$ and $V_{\mathrm{ECS},i}$ are the local concentration of free drugs and the local tissue ECS volume, respectively. $\epsilon_{\mathrm{ECS},i}$ is the volume fraction of local tissue ECS, and $V_{T,i}$ is local tissue volume. $V_{\mathrm{ECS},T}$ is the entire tissue ECS volume. The symbol ‘i’ refers to each control volume of the 3D computational mesh.

#### 2.6.2. Location of Distance Course

The tumor microenvironment can vary considerably from the infusion site to the brain tumor periphery, presenting a heterogenous asymmetrical distribution. To evaluate this spatial change, the location of the distance course ($\psi_{\mathrm{dis}}$) is applied, defined as

(17) $$\psi_{\mathrm{dis}}=\frac{\sum^{\;}\psi_{i}V_{T,i}}{\sum^{\;}V_{T,i}}\;\left(\mathrm{where}\;d_{i}=\mathrm{dis}\right)$$

in which ψ is the variable of interest. $d_{i}$ refers to the distance from the local tissue volume ($V_{T,i}$) to the infusion site, and dis is a given distance.

#### 2.6.3. Distribution Non-Uniformity

The non-uniformity of drug distribution is represented by a dimensionless number, NUN, which is defined as

(18) $$\mathrm{NUN}=\frac{\sum^{\;}\left|C_{\mathrm{FD},\mathrm{ECS},i}-C_{\mathrm{ECS},\mathrm{avg}}\right|V_{\mathrm{ECS},i}}{C_{\mathrm{FD},\mathrm{ECS},\mathrm{avg}}\sum^{\;}V_{\mathrm{ECS},i}}=\frac{\sum^{\;}\left|C_{\mathrm{FD},\mathrm{ECS},i}-C_{\mathrm{FD},\mathrm{ECS},\mathrm{avg}}\right|\epsilon_{\mathrm{ECS},i}V_{T,i}}{C_{\mathrm{FD},\mathrm{ECS},\mathrm{avg}}V_{\mathrm{ECS},T}}$$

NUN evaluates the spatial variation of the drug concentration. A lower value indicates a more uniform drug distribution.

#### 2.6.4. Effective Distribution Volume

The drug effective distribution volume indexes the treatment effectiveness ($V_{\mathrm{eff}}$) in which the local drug concentration is above the drug ${\mathrm{LD}}_{90}$, defined as

(19) $$V_{\mathrm{eff}}=\sum^{\;}V_{T,i}\;\left(\mathrm{where}\;C_{\mathrm{FD},\mathrm{ECS},i}\geq {\mathrm{LD}}_{90}\right)$$

## 3. Results

### 3.1. Model Validation

**Figure 4:**
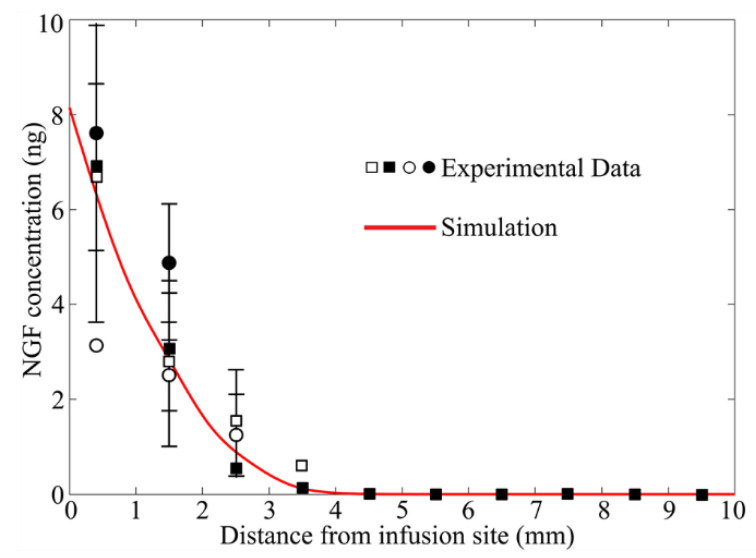
Comparison of experimentally measured and modelling predicted drug concentration upon CED infusion. Experimental data is extracted from Figure 3, left in Ref. [74].

The modelling predicted drug delivery outcomes are compared with experiments in Figure 4. The model in Section 2.1 was applied, while treating brain tissue as a porous medium with homogenous properties; the corresponding values are summarized in Table 3. The drug diffusivity in brain tissue is set as $1.8\times 10^{-11}\;m^{2}/s$ [73], and its elimination rate is $1.0\times 10^{-3}\;s^{-1}$ [73]. The modelling results agree with the experimental measurements [74], validating the model’s accuracy in predicting the drug delivery outcomes.

### 3.2. Baseline Study

The enhanced bulk flow of interstitial fluid is crucial for drug transport and accumulation in CED treatment. Due to antiangiogenesis, the dynamic change in tissue structure would reshape the hydraulic environment in the tumor and thereby alter the drug delivery outcomes. The infusion catheter is positioned at the site where the microvasculature is densest. Results of simulations using Patient 1 data are represented below. Results for Patient 2 show the same qualitative trends, which are included in Appendix A.

#### 3.2.1. Antiangiogenic Effect

**Figure 5:**
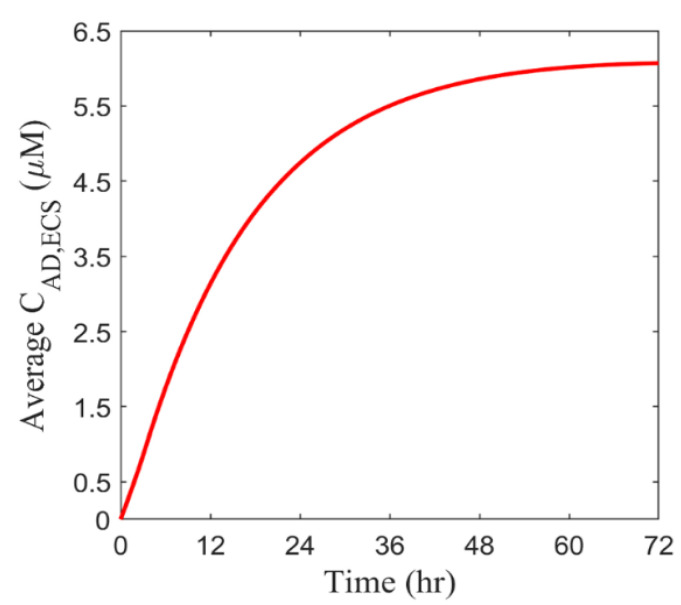
The spatial-averaged concentration of BEV in the brain tumor as a function of time.

Antiangiogenic drugs are simultaneously infused with cytotoxic drugs into the brain tumor in the combination therapy. Figure 5 represents the time course of BEV concentration in the entire tumor. BEV rapidly accumulates in the tumor on Day 1 upon the continuous infusion. However, this increase slows down as time proceeds and eventually tends to a constant level on Day 3. This is due to the dynamic equilibrium between the source term accounting for the drug supply by infusion and the sink term of drug elimination.

**Figure 6:**
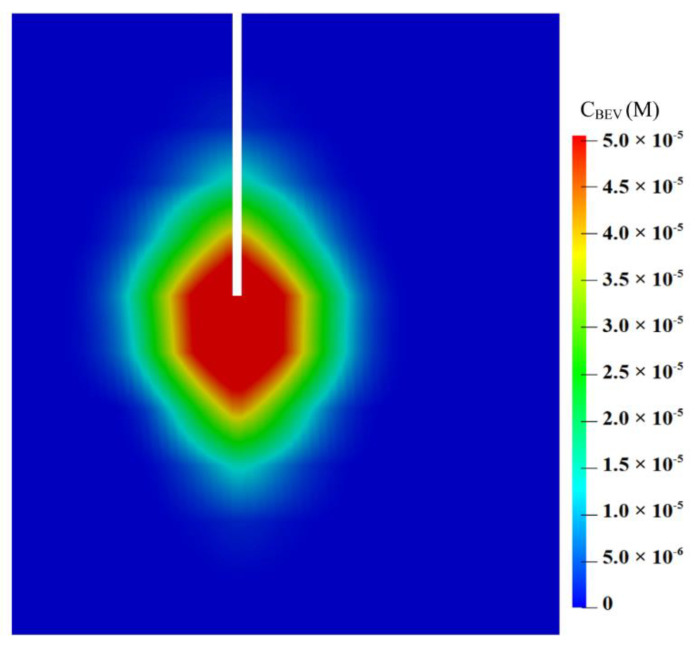
Spatial distribution of BEV on the vertical plane on Day 3.

The spatial distribution of BEV on Day 3 is shown on a vertical plane along the catheter track in Figure 6. The BEV concentration achieves its peak at the infusion site and decreases radially towards the domain boundary. Notably, although the infusion is highly directional, as pointed downward in Figure 6, the drugs can still transport along the catheter track and accumulate posteriorly at the infusion site. This is related to the drug diffusive transport that is determined by the concentration gradient between the infusion site and the tissue.

**Figure 7:**
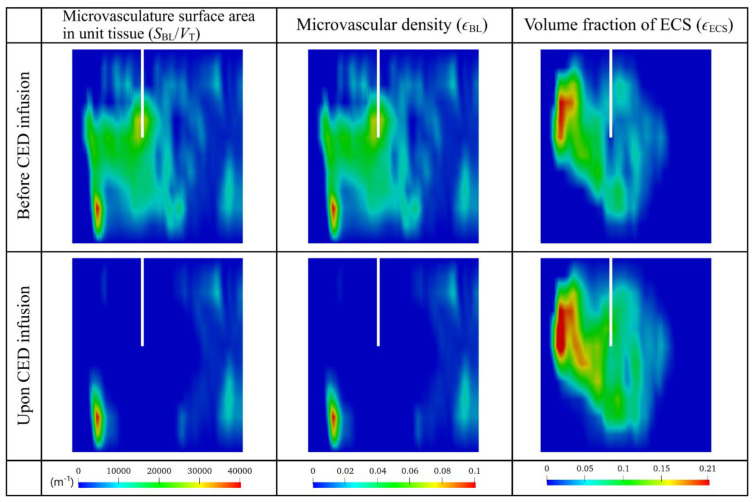
Distribution of tissue biological properties before and after the combination therapy. Results in the upper and lower panels are taken at time zero and Day 3, respectively.

The tumor’s biological properties before and after the combination therapy are compared on the same vertical plane in Figure 7. Results demonstrate the effectiveness of BEV in reducing the microvasculature surface area in unit tissue ($S_{\mathrm{BL}}/V_{T}$) and microvascular density ($\epsilon_{\mathrm{BL}}$). The antiangiogenic effect is not limited to the area in front of the catheter. Since BEV can transport deep in the tumor tissue as shown in Figure 6, microvasculature surface area and microvascular density around the catheter can also be reduced. This reduction can inhibit the drug loss by blood drainage, thereby retaining more drugs within the tumor for treatment. On the other hand, the volume fraction of extracellular space ($\epsilon_{\mathrm{ECS}}$) can consequently be enlarged, which would decrease the tissue resistance to the transport of cytotoxic drugs into the deep tumor region.

**Figure 8:**
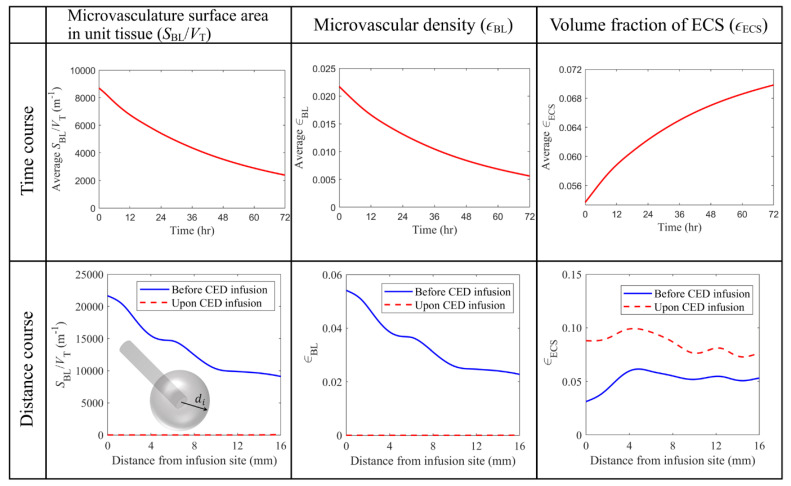
Comparison of tissue biological properties before and upon the combined CED infusion. The time course graphs in the top row are plotted using the average values in the entire brain tumor. The results taken on Day 3 are used to plot the distance course graphs in the bottom row.

The antiangiogenic effects of BEV on tumor properties are further quantitatively evaluated in Figure 8. The upper panel shows that $S_{\mathrm{BL}}/V_{T}$ and microvascular density decreases exponentially over time. The relatively slow changes on Day 3 indicate that the further prolongation of BEV infusion results in minor modifications to the tissue microstructure. Furthermore, an opposite trend can be observed for the volume fraction of ECS. This is because the space the blood vessels release is occupied by the interstitial fluid, becoming the extracellular space.

The tumor properties are represented in the lower panel as a function of the distance from the infusion site. The values are calculated for discrete voxels along a sphere surface described by the same distance of $d_{i}$. Comparisons show that BEV can successfully reduce the $S_{\mathrm{BL}}/V_{T}$ and microvascular density to a significantly low level in the entire tumor. Therefore, the volume fraction of ECS becomes higher throughout the brain tumor, easing the barrier for cytotoxic drugs to penetrate the tumor tissue.

#### 3.2.2. Interstitial Fluid Flow

**Figure 9:**
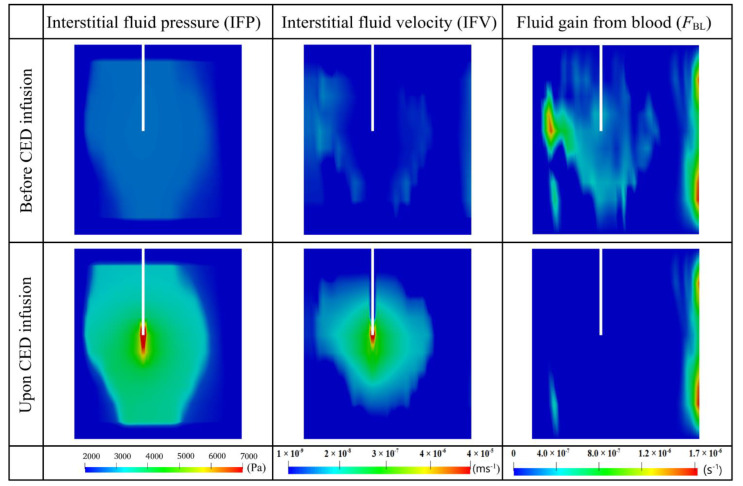
The distribution of interstitial fluid flow and fluid gain from the blood before and after the combined CED infusion. Results in the upper and lower panels are taken at time zero and Day 3, respectively.

Figure 9 compares the hydraulic environment before and after the combination therapy on the same vertical plane. Results show that IFP is higher in the tumor than in the surrounding normal tissue, consistent with the reported experiment finding [75]. Such high pressure is due to abnormal tumor properties, including high microvasculature surface area and blood vessel permeability, as shown in Table 3. However, as one moves towards the tumor periphery, IFP falls sharply at the interface between the tumor and normal tissue. More importantly, the CED infusion can override the original interstitial fluid flow to generate even higher pressure at the infusion site and raise the pressure in the entire tumor.

Relatively high IFV only occurs at the tumor-normal tissue interface due to the sharp fall of local IFP, which also promotes the outward convective transport of drugs from the tumor to its holding tissue. The velocity inside the tumor is low due to the even IFP distribution [17]. In contrast, CED infusion can accelerate the interstitial fluid flow in the entire brain tumor, with the most significant enhancement occurring at the infusion site. This rapid flow would improve convective drug transport for deep tissue penetration. Moreover, the comparison between the pre- and post-treatment denotes that the fluid leakage from blood can be significantly reduced in the combination therapy, due to the reduction of microvasculature density. This inhibited fluid leakage is beneficial for preventing the drug concentration from being diluted.

**Figure 10:**
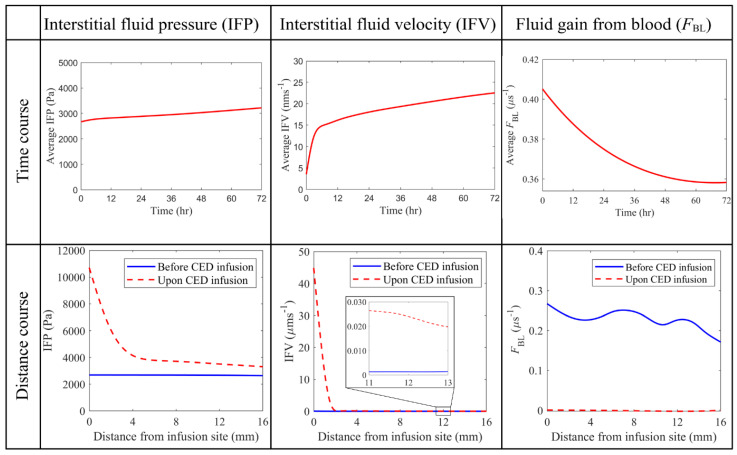
Comparison of hydraulic environments before and upon the combined CED infusion. The time course graphs in the top row are plotted using the average values in the entire brain tumor. The results taken on Day 3 are used to plot the distance course graphs in the bottom row.

Quantitative comparisons between the pre- and post-treatment hydraulic environments are given in Figure 10. IFP is uniform throughout the brain tumor before the combination treatment starts. This is because of the dynamic equilibrium reached between the blood pressure, osmotic pressure and IFP. In contrast, CED infusion would dramatically increase IFP around the infusion site to over $1.0\times 10^{5}\;\mathrm{Pa}$. Although this pressure experiences a rapid drop in approximately 4 mm away from the infusion site, the pressure in the whole tumor can still be raised. Thus, IFP throughout the tumor gradually increases over time in response to the continuous CED infusion. Similar trends can be found in the distance courses of IFV. The velocity reaches its peak at the infusion site and quickly decreases with the distance; however, it remains relatively higher in the deep tumor tissue (as seen in the zoomed portion). The time course of intratumoral spatial-averaged IFV exhibits two-phase changes. It increases linearly in the first 4~5 h after the CED infusion starts, while the increase slows down as the infusion continues. Moreover, modelling results show that the fluid leakage decreases once the antiangiogenic drugs are infused. The combination therapy can effectively reduce the fluid leakage from the blood in the entire tumor, as indicated by its distance course shown in the lower panel.

#### 3.2.3. Drug Concentration

The performance of the combination therapy using liposomal TMZ and BEV is evaluated by comparing to another three regimens under identical infusion settings. These include plain TMZ infusion, plain TMZ and BEV combined infusion, and liposomal TMZ infusion.

**Figure 11:**
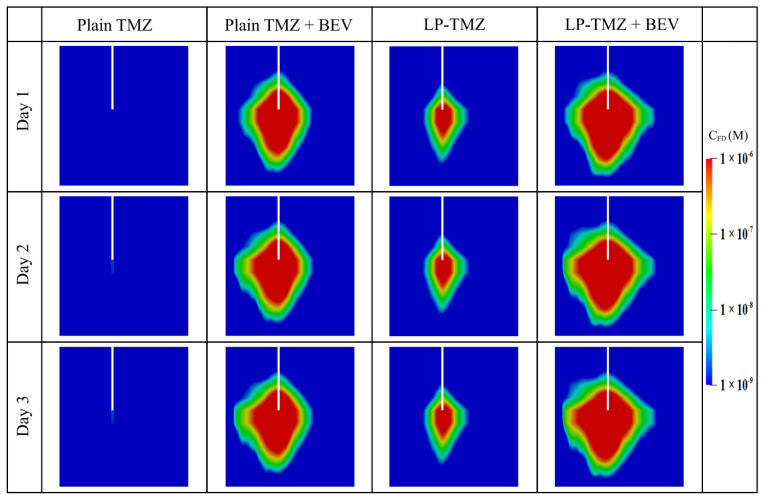
Spatial distribution of released free temozolomide at different time points.

Figure 11 compares the spatial distribution of free TMZ at different time points. The infusion of plain TMZ alone leads to minimal drug accumulation. This invisible drug concentration can be attributed to the fast drug elimination by blood drainage and bioreactions. Both the co-delivery with BEV and the application of liposomes can improve the delivery results. This is because the former inhibits blood drainage by reducing the microvasculature surface area, while the latter protects the drugs from unpreferred reactions. The most effective drug delivery can be obtained by co-delivering the liposomal TMZ with BEV. Moreover, the drug concentration presents similar distribution patterns since Day 2. This implies that the drug transport would reach a quasi-steady state after 48 h since the infusion starts; further prolongation of the infusion will contribute less to the distribution of drugs in the brain tumor.

Another set of simulations is performed based on the assumption of homogeneous tissue and drug properties. The microvascular density, tissue porosity and cell density are set uniform throughout the tumor and its surrounding tissue with their spatially-averaged values, respectively. At the same time, the rest infusion settings are kept identical. Results are shown in Appendix B. Drugs present a more symmetrical profile as compared to Figure 11. Moreover, the drug concentrations are overpredicted under this idealized condition, particularly for the plain TMZ infusion. These findings further demonstrate the role of a heterogeneous intratumoral environment in determining drug delivery outcomes.

**Figure 12:**
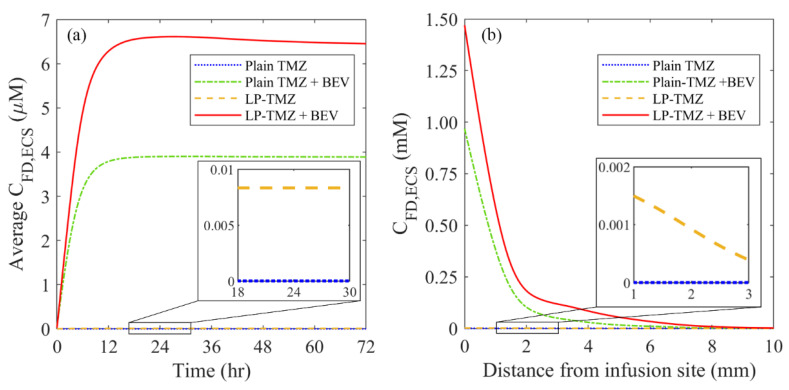
The (**a**) time courses, and (**b**) distance course of spatial-averaged free temozolomide concentration in the brain tumor under different dosing regimens. The results taken on Day 3 are used to plot the distance course graph.

The time courses of spatial-averaged free TMZ concentration are compared between different regimens in Figure 12a. The drug concentration increases sharply in the first 12 h and remains high over time. The maximum accumulation is achieved by co-delivery of liposomal TMZ with BEV, followed by the co-delivery of plain TMZ and BEV, liposomal TMZ infusion and plain TMZ infusion in sequence. A similar order can be found for the distance courses in Figure 12b. Furthermore, regardless of the dosing regimen, the drug concentrations reach their peak values at the infusion site and decrease rapidly with the increase in distance. The high TMZ concentrations can only be found in the area approximately 8 mm from the infusion site, indicating the delivery outcomes of CED are highly localized.

**Figure 13:**
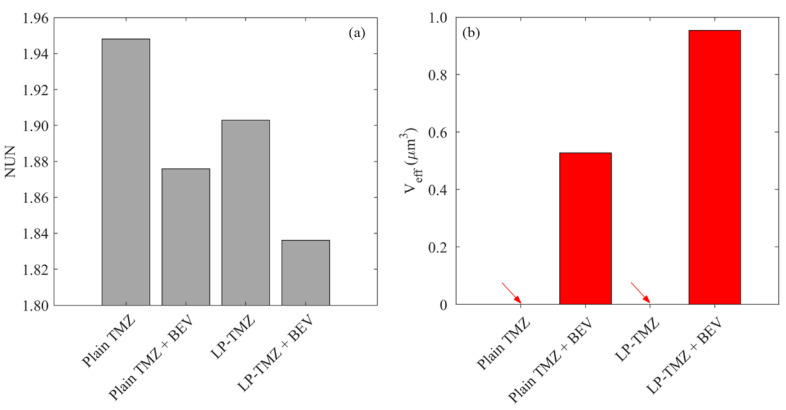
Comparisons of (**a**) drug distribution non-uniformity, and (**b**) effective distribution volume between different regimens in the brain tumor on Day 3.

The delivery outcomes of different regimens are further evaluated from the perspectives of drug distribution and treatment effectiveness in Figure 13. The results show that the direct infusion of plain TMZ leads to the most heterogeneous distribution in the brain tumor. Although the application of liposomes can homogenize drug distribution, this improvement is not as significant as co-delivery with BEV. The most uniform distribution can be found for the combined delivery of liposomal TMZ with BEV. The effective distribution volume varies distinctly between the regimens. Direct infusion of plain TMZ and liposomal TMZ fails to provide enough drug concentration to kill tumor cells effectively. In contrast, the treatment effectiveness can be improved by using antiangiogenic drugs. The liposomal TMZ and BEV combined delivery provides the most effective treatment.

### 3.3. Effect of Heterogeneous Microvasculature

The distribution of microvasculature can vary considerably throughout a brain tumor, depending on the tumor location and growth stage. This heterogeneous tumor characteristic would directly influence the combination therapy where BEV is used to reduce the microvascular density. A statistical analysis shows that the microvascular density ($\epsilon_{\mathrm{BL}}$) spans from $2.22\times 10^{-14}$ to $9.66\times 10^{-2}$ in this studied brain tumor. Therefore, three infusion sites with different $\epsilon_{\mathrm{BL}}$ are selected to examine the effects of microvasculature heterogeneity. The corresponding $\epsilon_{\mathrm{BL}}$ at these locations are $2.22\times 10^{-14}\;$(Case 1), $1.28\times 10^{-2}$ (Case 2) and $9.66\times 10^{-2}$ (Case 3), respectively.

**Figure 14:**
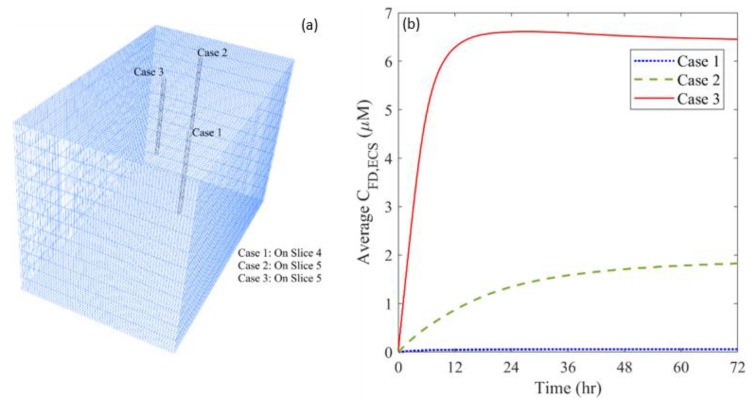
(**a**) Location of different infusion sites of plasma volume fraction, and (**b**) its impact on the time course of free temozolomide concentration in the brain tumor.

Figure 14 shows the time courses of free TMZ concentrations in the entire brain tumor for the infusion sites with different plasma volume fractions. The locations of the infusion site are shown in Figure 14a. After the treatment starts, TMZ begins to accumulate in the tumor ECS since drugs are continuously administrated. However, the accumulation rate varies. The concentration experiences a rapid increase in the first 24 h and remains at a relatively high level when the infusion site has the densest microvasculature. In contrast, infusing drugs into an area with fewer blood vessels results in a continuous increase of drug concentration over three days. Comparisons demonstrate that drug accumulation increases with the plasma volume fraction at the infusion site. The highest concentration can be achieved by placing the infusion catheter in the area with the highest $\epsilon_{\mathrm{BL}}$.

**Figure 15:**
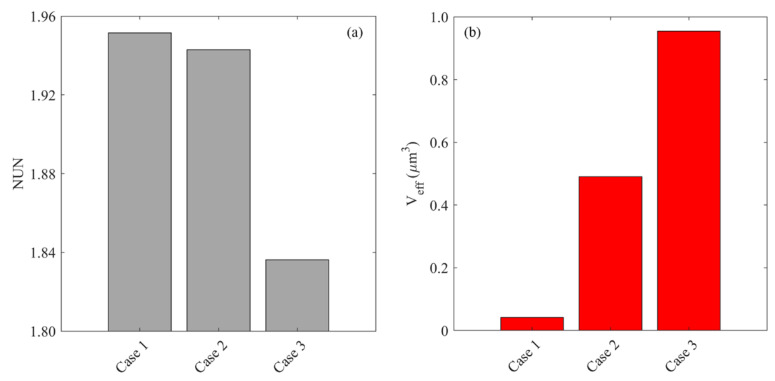
Impacts of the plasma volume fraction of infusion site on (**a**) drug distribution, and (**b**) treatment effectiveness in the brain tumor. The results are taken on Day 3.

Figure 15 compares the drug distribution and treatment effectiveness for the infusion sites with different plasma volume fractions. Results show that the distribution non-uniformity is inversely correlated to the local $\epsilon_{\mathrm{BL}}$. Infusing drugs in the area with a higher $\epsilon_{\mathrm{BL}}$ enables drugs to transport into deeper tumor tissue, leading to a more uniform distribution. This is because the antiangiogenic drugs can effectively reduce the drug loss to the blood and enlarge the extracellular space for drugs to transport. Consequently, the treatment effectiveness increases with the local $\epsilon_{\mathrm{BL}}$, since the deeper penetration allows the drugs to cover a larger tumor volume for effective cell killing.

### 3.4. Effect of Heterogeneous Cell Density

Cell density can be highly heterogeneous in a tumor, particularly for the tumor at an advanced stage and with a large dimension. This tumor property, reflected by the volume fraction of ICS ($\epsilon_{\mathrm{ICS}}$), affects the local drug cell uptake and the tumor ECS where the drugs transport. Given $\epsilon_{\mathrm{ICS}}$ is in the range of $5.808\times 10^{-1}$ to $9.99\times 10^{-1}$ in this examined tumor, three locations with different $\epsilon_{\mathrm{ICS}}$ are selected to examine its effects. The corresponding $\epsilon_{\mathrm{ICS}}$ at these three locations are $5.808\times 10^{-1}$ (Case 1), $8.144\times 10^{-1}$ (Case 2) and $9.99\times 10^{-1}$ (Case 3), respectively.

**Figure 16:**
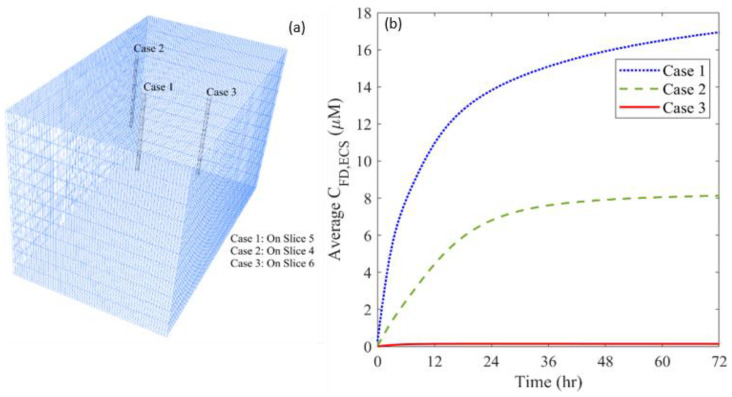
(**a**) Location of different infusion sites of cell volume fraction, and (**b**) its impact on the time course of free temozolomide concentration in the brain tumor.

The time courses of free TMZ concentration are compared for the infusion sites with different cell densities in Figure 16. Results show that the effectiveness of drug accumulation presents a negative relationship with the volume fraction of ICS at the infusion site. One possible reason is the low tissue porosity and microvascular density at the location where the cell density is high, resulting in less pronounced BEV-introduced improvement of drug transport.

**Figure 17:**
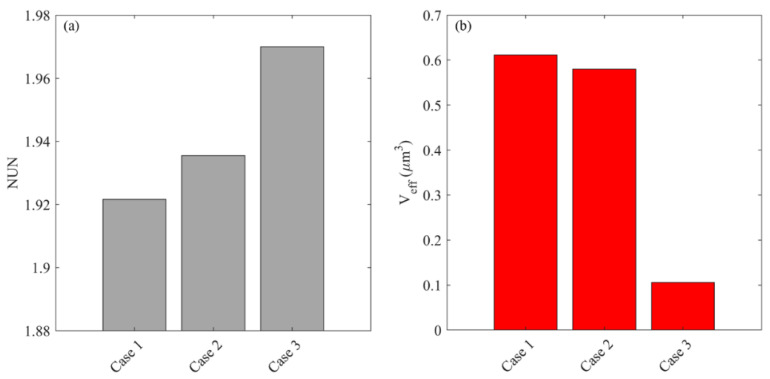
Impacts of cell volume fraction of infusion site on (**a**) drug distribution, and (**b**) treatment effectiveness in the brain tumor. The results are taken on Day 3.

The impact of cell volume fraction on drug distribution and treatment effectiveness is represented in Figure 17. The distribution of free TMZ would be more non-uniform when the infusion catheter is placed at the area with a high $\epsilon_{\mathrm{ICS}}$. Moreover, the effective distribution volume decreases with $\epsilon_{\mathrm{ICS}}$. These findings indicate that drug penetration is strongly limited, although infusing drugs to a location with higher cell density effectively kills the local tumor cells near the infusion site. This leads to a smaller tumor region with adequate drugs for treatment. Therefore, a better therapy could be achieved by infusing drugs to the location where the cell density is low.

## 4. Discussion

CED can effectively overcome the BBB by directly infusing drugs into the tumor. The rapid interstitial fluid flow improves the convective drug transport, enabling deep penetration into the tumor [5]. However, therapeutic effectiveness remains limited in clinical practice [6]. The intratumoral heterogeneous environment has been identified as one of the main limitations [7]. This modelling study demonstrates the effectiveness of co-delivery of cytotoxic drugs with antiangiogenic drugs for improving CED performance (Figure 11 and Figure 12). On the one hand, the decreased microvasculature surface area and density reduce fluid leakage from the blood (Figure 9), preventing dilution of drug concentrations (Equation (7)). On the other hand, drug elimination by blood drainage can be inhibited (Equation (7)). Since the previous study showed the CED-infused drugs would transport into the blood and accumulate in other tissues and organs [76], this inhibition is beneficial to retain more cytotoxic drugs within the tumor for therapy. Moreover, the delivery outcomes can be further improved by using liposomal cytotoxic drugs (Figure 13). This is because its low elimination rate allows the concentration of cytotoxic drugs to sustain at a relatively high level over time.

Given the heterogeneous characteristics of tumors, the location for positioning the catheter becomes critical. Treatment can be improved by infusing drugs at a site with denser microvasculature, as shown in Figure 15. As indicated in Figure 17, an inverse relationship is found between cell density and delivery outcomes; drug accumulation is more effective when the infusion catheter is placed at a site with a low cell density (Figure 16).

CED infusion would result in a significant increase in pressure at the infusion site. It is predicted to be greater than $1.0\times 10^{5}\;\mathrm{Pa}$, similar to the experimental measurements in the cat brains [77]. One must note that a brain tumor and its holding tissue can deform due to its soft nature in response to the CED infusion. Such high pressure can potentially cause tissue deformation and even tissue damage, consequently affecting drug delivery outcomes. Different models, including the poroelasticity model and hyper-viscoelasticity model, have been developed and applied to describe brain deformation [24,78,79,80]. However, due to the lack of support to obtain heterogeneous mechanical properties of tumor tissue, the CED-induced tumor deformation and its influence on the delivery outcomes are not addressed in this study. This impact can be examined by performing a parameter analysis in the following study. To avoid large tissue deformation, the infusion rate must be well controlled. It is recommended to be kept below 10 μL/min to avoid tissue damage. The modelling results also denote that the increase in pressure is highly localized. The average pressure across the tumor increases gradually during the infusion period. A multiphysics model incorporating fluid mechanics, tissue mechanics and fluid-solid interaction will need to be developed for in-depth analysis. This combination therapy involves complex physiological and physicochemical processes that are determined by various tissue and drug properties. A comprehensive parameter analysis using a simplified idealized model geometry allows for evaluating the role of each factor to identify the most influential ones. Results can reveal the underlying mechanisms in CED and benefit the treatment design for better effectiveness.

It is worth noting that the delivery outcomes of CED treatment strongly depend on multiple factors, including the catheter shape, infusion direction and injection profile. Future studies can be carried out to examine their impact for optimization. Results can not only improve the design of CED medical devices, but also contribute to the development of treatment protocols and guidelines.

The model predictive power for simulating drug delivery has been validated in Figure 4 and in several past studies. The IFP in the solid tumor was predicted to be 1500 Pa [31], which was well within the experimental range of 1064~3990 Pa [81,82]. The modelling results showed that the IFV in brain parenchyma was 0.65 μm/s [23]; this was in agreement with experimental measurements [49,83]. For CED, the model-predicted penetration depths of nanoparticles were consistent with the measurements from the in vivo experiments [40]. Similar comparisons can also be found for small molecules when infused into the gel phantom [84] and animal brain [73]. However, one must note that the model applied in this study is developed to capture the key physiological and physicochemical processes involved in the CED treatment. The model parameters listed in Table 3 and Table 4 are averaged and representative values from the literature. Therefore, the modelling results can only provide a qualitative trend of the delivery outcomes. Findings allow for identifying the importance of the examined influencing factors and determining the opportunities to improve the delivery outcomes. Medical imaging provides a non-invasive solution to obtain a realistic, patient-specific intratumoral environment, such as interstitial fluid flow and transport [85] and tissue anisotropy [35]. The image-derived information can be applied to improve the modelling accuracy.

This study involves several assumptions and limitations.

(a) The insertion of a catheter into the brain tissue has the potential to result in trauma and oedema. The fluid generated from damaged cells and the enhanced leakage from damaged blood vessels [86] can alter the interstitial fluid flow and cause swelling of brain tissue. However, there is a lack of accurate models to describe such a physiochemical process. Moreover, since oedema eventually disappears and the tissue properties can get back to normal levels [87,88], the effect of oedema is not considered. The mathematical model needs to be developed with support from in vivo experiments to describe this process.
(b) The antiangiogenic effect is reflected by the reduction of blood vessel diameter. Although blood vessels shirk significantly in response to the antiangiogenic drugs [66], both the vessel diameter and length can change [67]. Disappointingly, there is no such model to define the relationship between the changes in morphological characteristics of microvasculature and the antiangiogenic drug concentration. For improvement, results from microscale studies on the dynamic changes of blood vessels in antiangiogenic treatment will be needed for model development. In addition, modelling on the capillary level can be applied to predict the dynamic response of the microvascular network to the antiangiogenic drugs [89,90], which would shed light on the development of the combination therapy.
(c) Drug diffusivity can also be location-dependent in a brain tumor, subject to the local in vivo environment. This variation of drug property can also affect drug transport and accumulation. However, the diffusivity of each drug is assumed to be uniform in the brain tumor in this study due to the lack of relevant information that can be extracted from the applied medical images. This assumption can be relaxed by using diffusion-weighted MR images [91], where the signal intensity is related to the diffusivity of water molecules.
(d) Liposome cell uptake is ignored in this study owing to its complex nature. This process is controlled by several factors including particle size [92], ligands [93] and/or energy consumption [94,95]. A bespoken model needs to be developed to describe this process when focusing on a specific type of liposome.
(e) Only one catheter is placed in this study to examine the effectiveness of combination therapy in treating heterogeneous brain tumors. However, it is important to note that the cytotoxic drugs can penetrate a short distance from the infusion site; it is approximately 8 mm, as shown in Figure 12. This limited penetration depth results in highly localized cell killing, which is less effective for treating large tumors. In practice, it becomes more common to use multiple catheters [96] or a catheter with multiple injection-ports [97] simultaneously to enlarge the coverage of the drug to the tumor for better treatment outcomes. In this regard, the arrangement of catheters, the infusion directions and locations will be the key and worthy of in-depth study in the future.
(f) The infusate administrated into the brain tumor is able to push the soft tissue back to form backflow. Drugs in the backflow would transport along the track of the infusion catheter to the normal tissue, reducing the treatment effectiveness. The formation of backflow depends on the infusion rate, tumor location and more importantly, the local tissue properties. Given the difficulty to obtain the heterogeneous tissue mechanical properties from the applied medical images, this phenomenon is not simulated in this study. This limitation can be overcome by incorporating the tissue mechanics model, solid-fluid interaction and corresponding tissue properties [27,98].
(g) In the current study, the anti-angiogenetic and cytotoxic drugs were infused simultaneously inside the brain tumor due to the lack of clinical information regarding their infusion timings. Future studies in this direction should attempt to optimize the infusion schedule between antiangiogenetic and cytotoxic treatment to maximize the therapeutic output.

## 5. Conclusions

The combined CED of liposomal cytotoxic drugs and antiangiogenic drugs to a heterogeneous brain tumor has been studied by image-based mathematical modelling. Results show that the co-infusion of antiangiogenic drugs can effectively improve the delivery outcomes of cytotoxic drugs. The reduced microvascular density inhibits the fluid exchange between the blood and tissue as well as the drug loss due to blood drainage. Moreover, the location of the infusion site plays an important role in determining the performance of this combination therapy. Better treatment can be achieved by infusing drugs at denser microvasculature sites. Infusing the drug in a place with high cell density risks reducing its effectiveness against the entire tumor. Results obtained from this study can deepen the understanding of chemotherapy using CED. Please note that this study is based on the DCE-MRI data of two patients, thus further studies using a larger patient cohort will be needed to validate the results and to provide a reference for improving the treatment design.

## Figures and Tables

**Table 1:** T1-perfusion MR image acquisition protocol.

Parameter	Unit	Value
Repetition time (TR)	ms	6.27
Echo time (TE)	ms	3.0
Flip angle (θ)	deg	10
Field of view	mm	230×230
Slice thickness	mm	6.0
Reconstruction matrix	mm	256×256
Time of acquisition	min	2.15
Temporal resolution	sec	3.8

**Table 2:** Patient demographic characteristics.

Demographic Characteristics	Patient 1	Patient 2
Age (Years)	67	72
Gender	M	F
Tumor type	Glioblastoma	Meningioma
Newly diagnosed or recurrent tumor	Newly diagnosed	Newly diagnosed
Tumor grade	IV	I

**Table 3:** Biological properties of brain tumor and surrounding normal tissue.

Symbol	Parameter	Unit	Brain Tumor	Normal Tissue
$L_{\mathrm{BL}}$	Hydraulic conductivity of microvasculature wall	$m\cdot {\mathrm{Pa}}^{-1}\cdot s^{-1}$	$1.1\times 10^{-12}$ [23]	$1.4\times 10^{-13}$ [23]
${\left(S_{\mathrm{BL}}/V_{T}\right)}_{b}$	Baseline of blood vessel surface area per unit tissue volume	$m^{-1}$	$2.0\times 10^{4}$ [17]	$7.0\times 10^{3}$ [17]
$p_{\mathrm{BL}}$	Blood pressure	Pa	4610 [49]	4610 [49]
$\sigma_{\mathrm{BL}}$	Osmotic reflection coefficient for proteins in blood	−	0.82 [17]	0.91 [17]
$\pi_{\mathrm{BL}}$	Osmotic pressure of blood	Pa	3440 [49]	3440 [49]
$\pi_{\mathrm{ISF}}$	Osmotic pressure of interstitial fluid	Pa	1110 [49]	740 [49]
ρ	Interstitial fluid density	$\mathrm{kg}\cdot m^{-3}$	1000 [50]	1000 [50]
μ	Interstitial fluid viscosity	Pa·s	$7.8\times 10^{-4}$ [50]	$7.8\times 10^{-4}$ [50]
κ	Tissue hydraulic conductivity	$m^{2}$	$6.4\times 10^{-14}$ [23]	$6.5\times 10^{-15}$ [23]
α	Angiogenesis parameter	$s^{-1}$	$-1.85\times 10^{-6}$ [51]	$-1.85\times 10^{-6}$ [51]
β	Angiogenesis parameter	$s^{-1}$	$5.56\times 10^{-6}$ [51]	$5.56\times 10^{-6}$ [51]
γ	Angiogenesis parameter	$s^{-1}$	$-3.71\times 10^{-6}$ [51]	$-3.71\times 10^{-6}$ [51]

**Table 4:** Transport and pharmacological properties of drugs *.

Symbol	Parameter	Unit	Bevacizumab	Liposome	Temozolomide
MW	Molecular weight	$g\cdot {\mathrm{mol}}^{-1}$	$1.49\times 10^{5}$ [52]	−	$1.94\times 10^{2}$ [53]
$P_{\mathrm{TV}}$	Transvascular permeability	$m\cdot s^{-1}$	−	$5.8\times 10^{-8}$ (T) [54] 0 (N) [55]	$8.0\times 10^{-8}$ (T) [56] $4.3\times 10^{-8}$ (N) [56]
$D_{\mathrm{ECS}}$	Diffusivity in tissue ECS	$m^{2}\cdot s^{-1}$	$3.2\times 10^{-13}$ (T) ^‡^ [8] $4.9\times 10^{-14}$ (N) ^‡^ [8]	$1.5\times 10^{-13}$ (T) [57] $3.2\times 10^{-14}$ (N) [58]	$7.2\times 10^{-10}$ (T) [31] $3.4\times 10^{-10}$ (N) [59]
$K_{\mathrm{BC}}$	Binding constant of free drugs with proteins	−	−	−	0.18 [60]
$H_{\mathrm{CE}}$	CM/ECS partition coefficient	−	−	−	$1.5\times 10^{-2}$ [61]
$H_{\mathrm{IE}}$	ICS/ECS partition coefficient	−	−	−	1.0 [62]
$k_{a}$	Antiangiogenic rate	$M^{-1}\cdot s^{-1}$	1.12 ^‡^ [63]	−	−
$k_{e}$	Elimination rate due to reactions	$s^{-1}$	$1.2\times 10^{-5}$ [51]	−	$1.1\times 10^{-4}$ [60]
$k_{\mathrm{rel}}$	Drug release rate	$s^{-1}$	−	$6.4\times 10^{-4}$ [54]	−
${\mathrm{LD}}_{90}$	Drug dose that kills 90% of tumor cells	M	−	−	$3.9\times 10^{-5}$ [64]

* T and N stand for tumoral tissue and normal tissue, respectively. ^‡^ Estimated based on the drug molecular weight.

**Figure A1:**
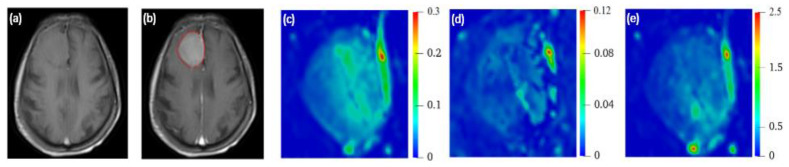
Tissue heterogeneous properties extracted from patient DCE-MR images. (**a**) Pre-contrast image, (**b**) post-contrast image, (**c**) tissue porosity $\epsilon_{\mathrm{ECS}}$, (**d**) microvascular density, $\epsilon_{\mathrm{BL}}$ and (**e**) $K^{\mathrm{trans}}$. The interface between the brain tumor and its holding tissue is marked in red in the post-contrast image.

**Figure A2:**
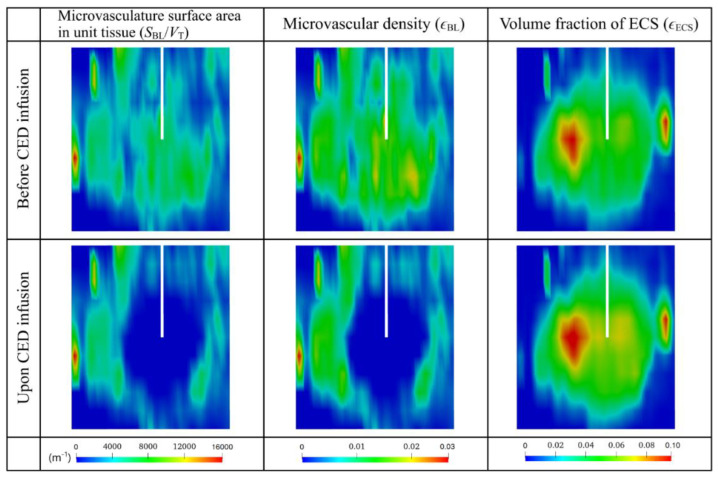
Distribution of tissue biological properties before and after the combination therapy. Results in the upper and lower panels are taken at time zero and Day 3, respectively.

**Figure A3:**
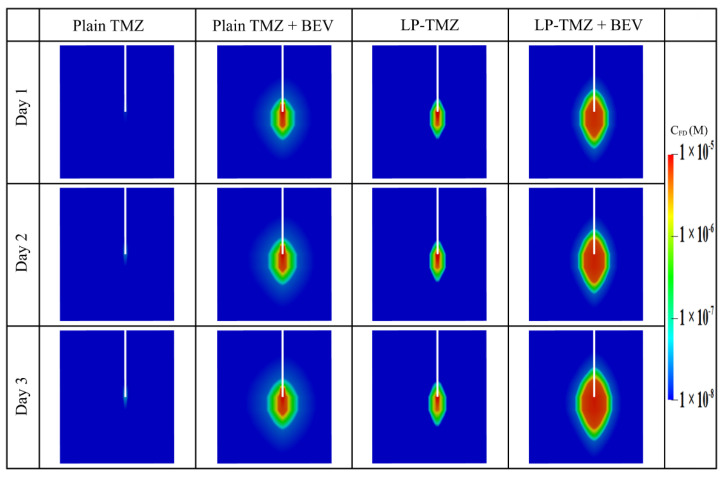
Spatial distribution of released free temozolomide at different time points.

**Figure A4:**
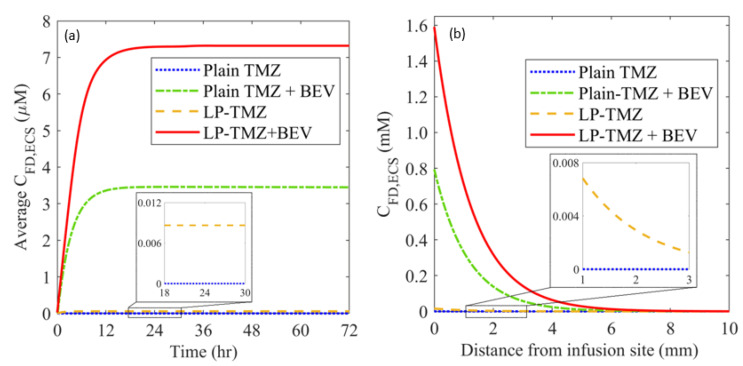
The (**a**) time courses, and (**b**) distance course of spatial-averaged free temozolomide concentration in the brain tumor under different dosing regimens. The results taken on Day 3 are used to plot the distance course graph.

**Figure A5:**
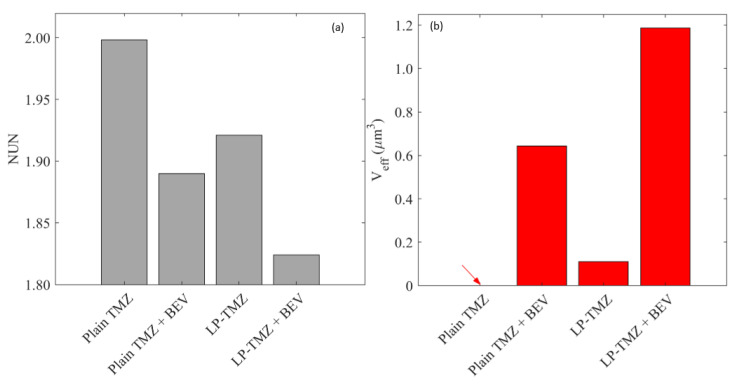
Comparisons of (**a**) drug distribution non-uniformity, and (**b**) effective distribution volume between different regimens in the brain tumor. The results are taken on Day 3.

**Figure A6:**
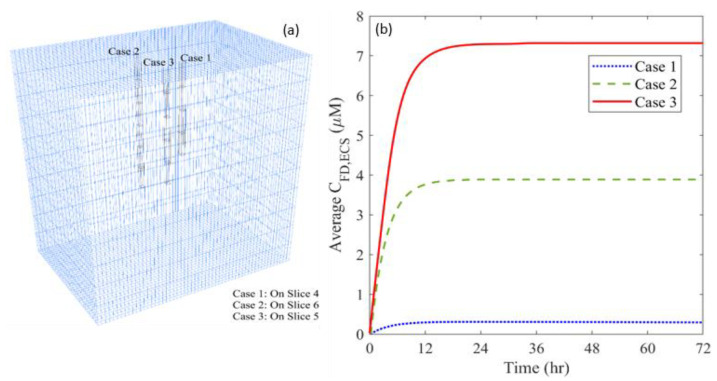
(**a**) Location of different infusion sites of plasma volume fraction, and (**b**) its impact on the time course of free temozolomide concentration in the brain tumor.

**Figure A7:**
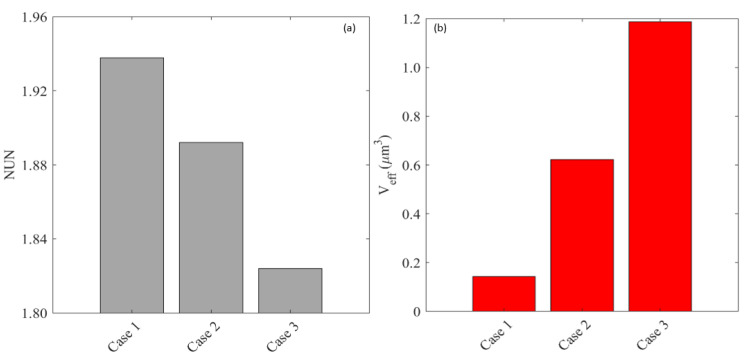
Impacts of the plasma volume fraction of infusion site on (**a**) drug distribution, and (**b**) treatment effectiveness in the brain tumor. The results are taken on Day 3.

**Figure A8:**
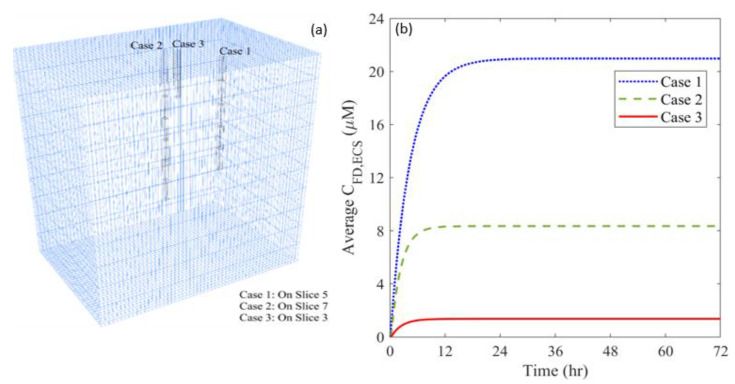
(**a**) Location of different infusion sites of cell volume fraction, and (**b**) its impact on the time course of free temozolomide concentration in the brain tumor.

**Figure A9:**
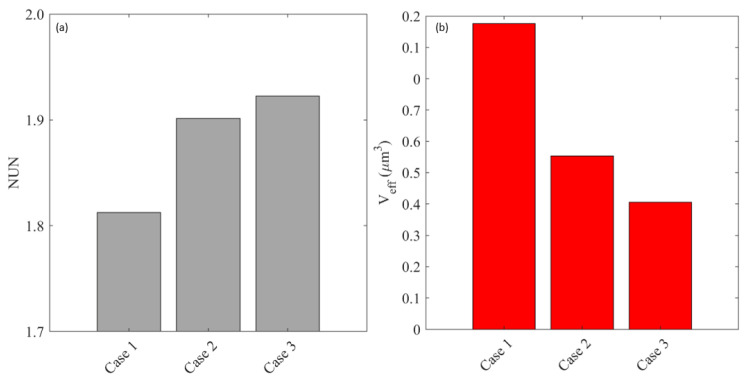
Impacts of cell volume fraction of infusion site on (**a**) drug distribution, and (**b**) treatment effectiveness in the brain tumor. The results are taken on Day 3.

**Figure A10:**
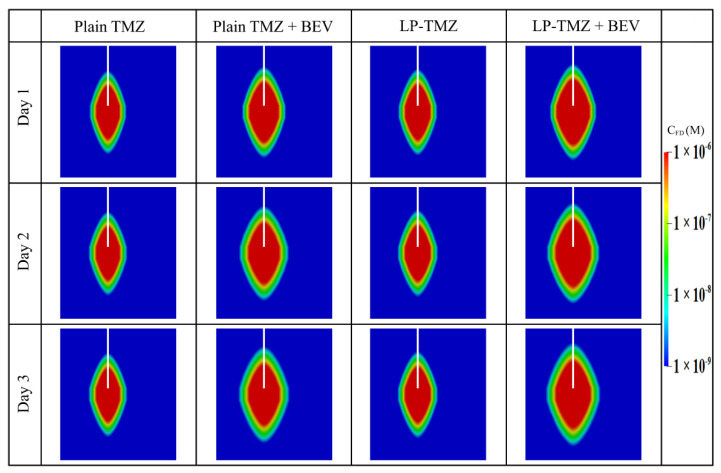
Spatial distribution of released free temozolomide at different time points.

## Data Availability

The data that support the findings of this study are available from the corresponding author, upon reasonable request.

## Appendix A. Modelling Using Patient 2′s Data

A set of simulations are performed using Patient 2′s data. The mathematical model, numerical method and imaging processing protocol are kept the same. Results are given in the following sections, supporting the conclusions.

### Appendix A.1. Tumor Geometry and Tissue Properties

The 3D geometry and tissue properties of Patient 2′s tumor and surrounding tissue are extracted from 12 slices of DCE-MR images using the methods given in Section 2.2. A representative slice (Slice 7) is shown in Figure A1.

### Appendix A.2. Baseline Study

The antiangiogenic drugs (BEV) and cytotoxic drugs are infused simultaneously into the brain tumor. The tissue biological properties before and after the CED infusion are compared in Figure A2 on a vertical plane along the infusion catheter. Results show that BEV can successfully reduce the microvascular surface area and microvascular density in the brain tumor and consequently increase the tissue porosity. These results are consistent with the ones shown in Figure 7.

The delivery outcomes using four different delivery scenarios are compared in Figure A3, including plain TMZ infusion, plain TMZ and BEV combined infusion, liposomal TMZ infusion, and liposomal TMZ and BEV combination infusion. It can be found that the infusion of free TMZ yields the worst drug profile, followed by liposomal TMZ infusion. The co-delivery with BEV can improve drug distribution; the best delivery outcomes are achieved by infusing BEV and liposomal TMZ.

The time course and distance course of spatially averaged concentration of free TMZ in the brain tumor are compared between the abovementioned four delivery scenarios, in Figure A4. Results demonstrate the advantages of combination therapy using BEV and liposomal TMZ in improving drug accumulation and penetration.

The distribution heterogeneity of free TMZ is compared between the four delivery scenarios in Figure A5a. The combination delivery of BEV and liposomal TMZ leads to the most homogeneous distribution pattern; whereas drugs would concentrate in a limited region when free TMZ is mono-infused. Similarly, to the results in Figure 13, the largest effective distribution volume can be found for the co-infusion of BEV and liposomal TMZ, as denoted in Figure A5b. The only difference is the infusion of liposomal TMZ that can also provide effective cell killing, which is not shown in Figure 13. However, this effective distribution volume is still much lower than that of combination therapy.

### Appendix A.3. Effect of Microvascular Density

Three infusion sites with different $\epsilon_{\mathrm{BL}}$ are selected in the second brain tumor, as $4.6\times 10^{-4}$ (Case 1), 0.01 (Case 2) and 0.04 (Case 3), respectively. The locations of these infusion sites are illustrated in Figure A6a. Results in Figure A6b denote that the accumulation of free TMZ is positively related to the local microvascular density, which is consistent with the findings obtained from Figure 14.

The delivery outcomes are evaluated in terms of NUN and $V_{\mathrm{eff}}$ in Figure A7. Simulations show the free TMZ would present a more homogenous distribution pattern to cover a larger tumor area for effective therapy when the infusion catheter is located at a higher microvascular density site. This agrees with the results in Figure 15.

### Appendix A.4. Effect of Cell Density

Three infusion sites with different $\epsilon_{\mathrm{ICS}}$ are selected in the second brain tumor, as 0.878 (Case 1), 0.9603 (Case 2) and 0.9872 (Case 3), respectively. The places where the infusion catheter is located in the brain tumor are shown in Figure A8a. Results in Figure A8b denote that the accumulation of free TMZ is inversely correlated to the local cell density at the infusion site.

The evaluation of drug distribution and potential treatment effectiveness of the three cases is shown in Figure A9. It can be found that infusing drugs at the location with the highest cell density results in the most non-uniform distribution and the worst cell killing. These are consistent with the findings shown in Figure 17.

## Appendix B. Impact of the Assumption of Homogeneous Tissue Properties

In this study, it is assumed that the tissue properties are homogeneously distributed in the brain tumor and normal tissue, respectively. These properties include the microvascular density, tissue porosity, cell density and microvasculature surface area per tissue volume. The modelling is based on Patient 1′s data, and the spatially averaged values of the abovementioned properties are applied. The rest of the properties’ values are the same as listed in Table 3 and Table 4.

Figure A10 represents the free TMZ distribution under the four delivery scenarios at different time points. The drugs present a more symmetrical distribution compared to the results in Figure 10. Furthermore, the drug concentration is also overestimated. This is highly associated with the non-uniformly distributed microvasculature, which can rapidly eliminate the drugs before they penetrate deep into the tissue. This comparison demonstrates the impact of the tumor heterogeneous environment on drug transport.
